# Supplementary material for: An open-source framework for large-scale, flexible evaluation of biomedical text mining systems
Source: J Biomed Discov Collab. 2008 Jan 29;3:1. doi: 10.1186/1747-5333-3-1 (PMC2276192; doi:10.1186/1747-5333-3-1)
Supplement: Additional file 1 — Evaluation platform source code. The additional file contains the software evaluation framework discussed in this paper. The most current release can be downloaded from SourceForge.net [1]. See the accompanying README file for instructions on installation and use. Also included in the distribution is a collection of UIMA wrappers for some commonly used BioNLP tools and annotated corpora. [file 1747-5333-3-1-S1.zip › bionlp_uima_repository-1.0.1/docs/api/edu/uchsc/ccp/util/nlp/document/GenericDocument.html]

GenericDocument (Center for Computational Pharmacology Util/UIMA API)


|  |  |  |
| --- | --- | --- |
| **Overview** \| **Package** \| **Class** \| **Tree** \| **Deprecated** \| **Index** \| **Help** | |  |
| **PREV CLASS**   NEXT CLASS | **FRAMES**    **NO FRAMES**     **All Classes** |  |
| SUMMARY: NESTED \| FIELD \| CONSTR \| METHOD | DETAIL: FIELD \| CONSTR \| METHOD |  |


---


## edu.uchsc.ccp.util.nlp.document Class GenericDocument

```
java.lang.Object
  edu.uchsc.ccp.util.nlp.document.GenericDocument
```

---

``` public class GenericDocument extends Object ```

---

**Constructor Summary**

`GenericDocument()`

`GenericDocument(String documentID)`


| **Method Summary** | |
| --- | --- |
| `void` | `addAnnotation(TextAnnotation annotation)` |
| `void` | `addDocumentSection(DocumentSection ds)` |
| `void` | `addOtherDocumentID(String idType, String id)`             Insert a new document ID (other than the primary ID) |
| `void` | `addSecondaryDocumentID(String secondaryDocID)`             **Deprecated.** |
| `String` | `getAbstractText()`             **Deprecated.** |
| `List<TextAnnotation>` | `getAnnotations()` |
| `int` | `getDocumentCollectionID()` |
| `String` | `getDocumentID()` |
| `List<DocumentSection>` | `getDocumentSections()` |
| `String` | `getDocumentText()` |
| `Map<String,String>` | `getOtherDocumentIDs()`             Retrieve any other identifiers associated with this document |
| `List<String>` | `getSecondaryDocumentIDs()`             **Deprecated.** |
| `String` | `getTitleText()`             **Deprecated.** |
| `void` | `printDocument(PrintStream ps)` |
| `void` | `setAbstractText(String abstractText)`             **Deprecated.** |
| `void` | `setAnnotations(List<TextAnnotation> annotations)` |
| `void` | `setDocumentCollectionID(int documentCollectionID)` |
| `void` | `setDocumentID(String documentID)` |
| `void` | `setDocumentSections(List<DocumentSection> documentSections)` |
| `void` | `setDocumentText(String documentText)` |
| `void` | `setSecondaryDocumentIDs(List<String> secondaryDocumentIDs)`             **Deprecated.** |
| `void` | `setTitleText(String titleText)`             **Deprecated.** |

**Methods inherited from class java.lang.Object**

`clone, equals, finalize, getClass, hashCode, notify, notifyAll, toString, wait, wait, wait`

**Constructor Detail**

### GenericDocument

```
public GenericDocument(String documentID)
```

---


### GenericDocument

```
public GenericDocument()
```


**Method Detail**

### getOtherDocumentIDs

```
public Map<String,String> getOtherDocumentIDs()
```

:   Retrieve any other identifiers associated with this document

    :   **Returns:**

---


### addOtherDocumentID

```
public void addOtherDocumentID(String idType,
                               String id)
```

:   Insert a new document ID (other than the primary ID)

    :   **Parameters:**: `idType` -: `id` -

---


### getAnnotations

```
public List<TextAnnotation> getAnnotations()
```

---


### setAnnotations

```
public void setAnnotations(List<TextAnnotation> annotations)
```

---


### addAnnotation

```
public void addAnnotation(TextAnnotation annotation)
```

---


### getAbstractText

```
@Deprecated
public String getAbstractText()
```

:   **Deprecated.**

---


### setAbstractText

```
@Deprecated
public void setAbstractText(String abstractText)
```

:   **Deprecated.**

---


### getTitleText

```
@Deprecated
public String getTitleText()
```

:   **Deprecated.**

---


### setTitleText

```
@Deprecated
public void setTitleText(String titleText)
```

:   **Deprecated.**

---


### getDocumentID

```
public String getDocumentID()
```

---


### setDocumentID

```
public void setDocumentID(String documentID)
```

---


### getDocumentSections

```
public List<DocumentSection> getDocumentSections()
```

---


### addDocumentSection

```
public void addDocumentSection(DocumentSection ds)
```

---


### setDocumentSections

```
public void setDocumentSections(List<DocumentSection> documentSections)
```

---


### getDocumentText

```
public String getDocumentText()
```

---


### setDocumentText

```
public void setDocumentText(String documentText)
```

---


### getDocumentCollectionID

```
public int getDocumentCollectionID()
```

---


### setDocumentCollectionID

```
public void setDocumentCollectionID(int documentCollectionID)
```

---


### getSecondaryDocumentIDs

```
@Deprecated
public List<String> getSecondaryDocumentIDs()
```

:   **Deprecated.**

---


### setSecondaryDocumentIDs

```
@Deprecated
public void setSecondaryDocumentIDs(List<String> secondaryDocumentIDs)
```

:   **Deprecated.**

---


### addSecondaryDocumentID

```
@Deprecated
public void addSecondaryDocumentID(String secondaryDocID)
```

:   **Deprecated.**

---


### printDocument

```
public void printDocument(PrintStream ps)
```


---


|  |  |  |
| --- | --- | --- |
| **Overview** \| **Package** \| **Class** \| **Tree** \| **Deprecated** \| **Index** \| **Help** | |  |
| **PREV CLASS**   NEXT CLASS | **FRAMES**    **NO FRAMES**     **All Classes** |  |
| SUMMARY: NESTED \| FIELD \| CONSTR \| METHOD | DETAIL: FIELD \| CONSTR \| METHOD |  |


---
